# Vascular loops in the anterior inferior cerebellar artery, as identified by magnetic resonance imaging, and their relationship with otologic symptoms*

**DOI:** 10.1590/0100-3984.2015.0069

**Published:** 2016

**Authors:** Luiz de Abreu Junior, Cristina Hiromi Kuniyoshi, Angela Borri Wolosker, Maria Lúcia Borri, Augusto Antunes, Vanessa Kiyomi Arashiro Ota, Daniela Uchida

**Affiliations:** 1 PhD, MD, Radiologist in the Grupo Fleury at the Hospital São Luiz/Rede D'Or, São Paulo, SP, Brazil.; 2 MD, Radiologist in the Grupo Fleury at the Hospital São Luiz/Rede D'Or, São Paulo, SP, Brazil.; 3 MD, Radiologist at Axial Medicina Diagnóstica, Belo Horizonte, MG, Brazil.; 4 Biomedical Professional, Postdoctoral Student in the Department of Psychiatry at the Escola Paulista de Medicina da Universidade Federal de São Paulo (EPM-Unifesp), São Paulo, SP, Brazil.

**Keywords:** Arteries/anatomy & histology, Nerve compression syndromes, Magnetic resonance imaging, Tinnitus, Hearing loss, Vertigo

## Abstract

**Objective:**

To use magnetic resonance imaging to identify vascular loops in the anterior inferior cerebellar artery and to evaluate their relationship with otologic symptoms.

**Materials and Methods:**

We selected 33 adults with otologic complaints who underwent magnetic resonance imaging at our institution between June and November 2013. Three experienced independent observers evaluated the trajectory of the anterior inferior cerebellar artery in relation to the internal auditory meatus and graded the anterior inferior cerebellar artery vascular loops according to the Chavda classification. Kappa and chi-square tests were used. Values of *p* < 0.05 were considered significant.

**Results:**

The interobserver agreement was moderate. Comparing ears that presented vascular loops with those that did not, we found no association with tinnitus, hearing loss, or vertigo. Similarly, we found no association between the Chavda grade and any otological symptom.

**Conclusion:**

Vascular loops do not appear to be associated with otoneurological manifestations.

## Abstract (pt)

**Objetivo:**

O objetivo deste estudo foi avaliar e analisar, por ressonância magnética, a presença e o tipo de alça vascular da artéria cerebelar anteroinferior e sua associação com sintomas otológicos.

**Materiais e Métodos:**

Selecionamos 33 adultos com queixas otológicas que realizaram ressonância magnética em nosso serviço, entre junho e novembro de 2013. Três observadores experientes, independentes, avaliaram o trajeto da artéria cerebelar anteroinferior e sua relação com o conduto auditivo interno, classificando os tipos de alça vascular segundo a classificação de Chavda. Os testes de kappa e de qui-quadrado foram utilizados e o índice de significância adotado foi *p* < 0,05.

**Resultados:**

O coeficiente kappa entre diferentes observadores demonstrou concordância moderada. Comparando as orelhas que apresentavam alça vascular com as que não apresentavam, não encontramos associação com zumbido, hipoacusia ou tontura. Semelhantemente, comparando os diferentes graus da classificação de Chavda com os sintomas relatados, não encontramos associação estatisticamente significante.

**Conclusão:**

Concluímos que não há associação entre a presença ou o tipo de alça vascular e sintomas otoneurológicos.

## INTRODUCTION

Common otologic symptoms include tinnitus (the perception of sounds in the absence of external stimuli), hearing loss, and dizziness. The estimated prevalence of these symptoms in the Brazilian population is approximately 22% for tinnitus^(1)^, 9% for hearing loss^(2)^, and 42% for dizziness^(3)^, and those rates increase with advancing age.

Although various diseases are associated with otologic symptoms, the cause is not always identified. In some cases, it is believed that the etiology involves a vascular loop in the anterior inferior cerebellar artery (AICA), insinuating itself into the internal auditory meatus.

The term vascular compression syndrome, which refers to a group of diseases caused by direct contact between a blood vessel and a cranial nerve, was introduced by McKenzie in 1936 and popularized by Jannetta in 1975^(4,6)^. The prototype of this syndrome, hemifacial spasm, was first described in 1875, when a vertebral artery aneurysm was found to be compressing the facial nerve of a patient^(7)^. The concept has since been expanded to explain diseases related to various cranial nerves.

Jannetta et al., for example, suggested that redundant arterial loops could interfere with the vestibulocochlear nerve (eighth cranial nerve), resulting in otologic symptoms^(8)^. Although numerous articles have focused on this condition, the existence of vascular compression syndromes continues to be questioned.

The objective of the present study was to evaluate and analyze, through magnetic resonance imaging (MRI), the presence of vascular loops and their association with the otologic profile.

## MATERIALS AND METHODS

We selected 33 adults with otologic complaints who underwent MRI at our facility between June and November of 2013. These patients completed a questionnaire and gave written informed consent. The sample comprised 21 women (63.6%) and 12 men (36.4%). The mean age was 51.1 ± 17.3 years (range, 21-83 years).

We evaluated the trajectory of the AICA in relation to the internal auditory meatus through a T2-weighted sequence, using the three-dimensional driven equilibrium technique, which increases the contrast between the cerebrospinal fluid in the subarachnoid space and the cranial nerves. The scans were obtained in 1.0 T and 1.5 T MRI scanners (Gyroscan-NT and Intera, respectively; Philips, Best, the Netherlands). Three radiologists, each with more than five years of experience, graded the vascular loops according to the Chavda classification^(8)^. The raters were blinded to the sidedness of the otologic symptom.

The Chavda classification grades the vascular loops in the AICA as follows^(8)^: grade I - when an AICA vascular loop borders the internal auditory meatus (internal acoustic pore); grade II - when the loop insinuates itself into the internal auditory meatus but occupies 50% or less of the canal; or grade III - when the loop occupies more than 50% of the canal.

We used the chi-square test to determine whether the presence of a vascular loop was associated with tinnitus, hearing loss, or dizziness The level of significance adopted was 0.05. The kappa test was used in order to characterize the concordance between the examiners as to the type of vascular loop identified.

## RESULTS

The frequencies of otologic symptoms (tinnitus, hearing loss, and dizziness) are listed in Table 1. Of the 33 patients evaluated, 14 (42.4%) had complained of tinnitus (in either ear), 14 (42.4%) had reported hearing loss, and 25 (75.8%) had complained of dizziness.

**Table 1:** Tinnitus, hearing loss, and dizziness in the sample studied.

Otologic symptom	Frequency
Tinnitus	
Right ear only	4
Left ear only	7
Both ears	3
Total ears with tinnitus	17 (25.8%)
Total ears without tinnitus	49 (74.2%)
Hearing loss	
Right ear only	5
Left ear only	7
Both ears	2
Total ears with hearing loss	16 (24.2%)
Total ears without hearing loss	50 (75.8%)
Dizziness	
Total cases with dizziness	25 (75.8%)
Total cases without dizziness	8 (24.3%)

To determine the Chavda grade, we first calculated the interobserver agreement. The kappa values are shown in Table 2.

**Table 2:** Interobserver agreement (kappa values) among the three evaluators.

	Evaluator 1	Evaluator 2	Evaluator 3
Evaluator 1	1	0.746	0.684
Evaluator 2	0.746	1	0.628
Evaluator 3	0.684	0.628	1

**Figure 1:**
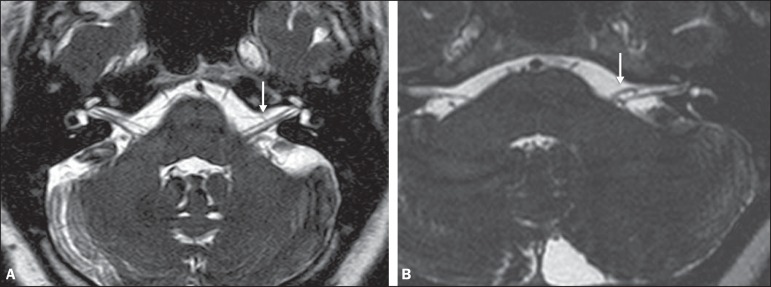
Chavda grade I vascular loops in the AICA. Note that the vessel runs alongside the internal auditory meatus of the left ear, without extending to the inside (arrows).

**Figure 2:**
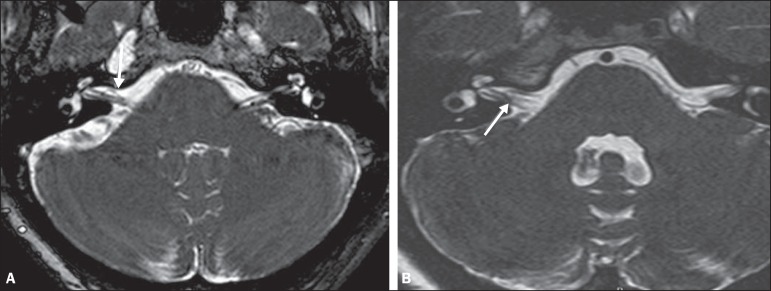
Examples of Chavda grade II vascular loops in the AICA. The vascular structure extends to the interior of the internal auditory meatus but occupies 50% or less of the canal (arrows).

**Figure 3:**
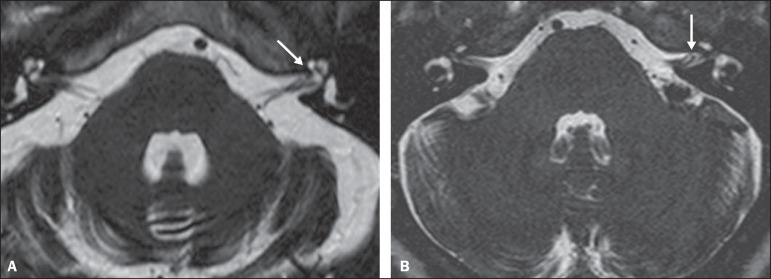
Examples of Chavda grade III vascular loops in the AICA. The vessel occupies more than 50% of the canal of the internal auditory meatus, reaching its basal portion (arrows).

Examples of MRI scans in which Chavda grade I, II, and III vascular loops were found are shown in Figures 1, 2, and 3, respectively.

To evaluate interobserver agreement regarding the grading of the vascular loops, we separated the data by sidedness and considered the Chavda classification concordant if at least two of the three evaluators were in agreement. In this analysis, a total of 66 ears were evaluated. Two of those were excluded for presenting different classifications among the three evaluators. Therefore, the final sample comprised 64 ears.

Of the 64 ears evaluated, 28 (43.75%) presented no vascular loop, 31 (48.44%) presented a grade I Chavda vascular loop and 5 (7.81%) presented a grade II Chavda vascular loop.

The results of the chi-square tests comparing the occurrence of otologic symptoms (tinnitus, hearing loss, and dizziness) with the presence of vascular loops and with the Chavda classification are shown in Table 3. The p values indicate that the otoneurological symptoms were no associated with the presence or type of vascular loop.

**Table 3:** Results of the chi-square tests comparing the occurrence of otologic symptoms with the presence of vascular loops and with the Chavda classification.

	No vascular loop	Chavda grade I	Chavda grade II	χ^2^	*P*
Tinnitus	Yes = 8	Yes = 7	Yes = 1	0.354	0.838
	No = 20	No = 24	No = 4		
Hearing loss	Yes = 8	Yes = 6	Yes = 1	0.732	0.693
	No = 20	No = 25	No = 4		
Dizziness	Yes = 22	Yes = 23	Yes = 3	1.132	0.568
	No = 5	No = 7	No = 2		

Level of significance: p < 0.05.

We also evaluated the symptom groups in the absence or presence of a (Chavda grade I or II) vascular loop. However, no statistically significant results were obtained (tinnitus: χ^2^ = 0.339, p = 0.561; hearing loss: χ^2^ = 0.731, p = 0.393; dizziness: χ^2^ = 0.451, p = 0.502).

## DISCUSSION

Our results indicate that there is association between otologic symptoms and the presence or type of vascular loop in the AICA. We stratified the results by the ear affected and considered Chavda classification concordant only if at least two of the three evaluators were in agreement. These findings corroborate the bulk of the data in the literature^(5,9,11)^.

The concept of vascular compression syndromes was popularized by Jannetta^(6)^, who reported a reduction in dysfunctional hyperactivity of the eighth cranial nerve after using microsurgery to separate the nerve from a blood vessel, supporting the theory that a vascular loop is an etiological factor of dysfunction. On the basis of that study, various other researchers have attempted to establish a relationship between vascular compressions and a number of clinical conditions, such as hemifacial spasm and trigeminal neuralgia^(4)^. However, controversy remains regarding the pathophysiology of these conditions. It has been suggested that chronic compression is responsible for regional nerve demyelination or that disturbances in the distribution of blood flow result in reduced vascular perfusion of nerves, either of which could explain the clinical profiles of vascular compression syndromes^(12,15)^.

It is of note that new, highly sensitive MRI techniques have made it possible to investigate the relationship between intracranial vessels and nerves in a non-invasive manner. Volumetric sequences with strong T2 weighting (constructive interference in steady state imaging, fast imaging employing steady-state acquisition, and balanced fast-field echo imaging) present advantages over conventional angiographic examinations, given that, in addition to being non-invasive (not involving the use of contrast), the former allow the assessment of the blood vessels, as well as of the nerve in question and the harmonic or potentially pathological relationship between the two^(16,17)^.

Although the concept of vascular compression has been widely accepted for hemifacial spasm and trigeminal neuralgia, its relationship with otologic symptoms such as tinnitus, hearing loss, and dizziness is not yet clear. Otologic complaints are relatively common in daily life, and it is not uncommon to see cases in which the cause of such complaints is not identified; for many of those cases, vascular compression has been considered an etiological factor^(18)^.

Makins et al. found no significant differences between ears with clinical signs and symptoms and healthy (asymptomatic) ears regarding the presence of vascular loops, suggesting that the presence of of vascular loop on MRI is not pathological, per se, and can be viewed more as a normal anatomic finding^(5)^. Similarly, Grocoske et al. found that the presence of a neurovascular conflict involving the eighth cranial nerve on MRI scans could not, in and of itself, explain the otoneurological signs and symptoms observed in the subjects assessed^(9)^. In the present study, we also found no association between the presence or type of vascular loop seen on the MRI scans and the otoneurological signs and symptoms reported.

Post-mortem studies have shown that vascular loops in the AICA occur within the internal auditory meatus in 12.3% of human temporal bones^(11,19)^. However, that might differ from the reality *in vivo*, because changes can occur as a result of the formaldehyde fixation process^(20)^. Therefore, the mere presence of vascular loops in the AICA might not be an indication that there is vascular compression of the eighth cranial nerve. It should be borne in mind that it can represent a simple anatomic variation. It is of note that most of the patients of our sample (*n* = 36) had vascular loops that were classified as Chavda grade I or II. In a study conducted by Gultekin et al., 72% of the controls had vascular loops in the AICA involving the internal auditory meatus (Chavda grade I)^(10)^. However, the data are still controversial, and some studies that have indicated that there is in fact an association between vascular loops and otologic symptoms^(6,21,24)^.

The discrepancies among the results can be explained, at least in part, by differences among observers, which can skew the results of the estimates and the application of different types of classification. In the present study, we used three evaluators and found that the interobserver agreement, as estimated by the kappa index, was moderate to substantial for the classification of vascular loops. Some of the inconsistencies can be explained by the difficulty in differentiating between the successive types of vascular loops according to the Chavda classification. In view of that, we included in our analysis only the values which were concordant between at least two observers, reducing the risk of observer bias. Further studies would be useful in order to detail the relationship between the vascular structure and the nervous system, perhaps generating new classifications that increase the power to discriminate between truly pathological cases and those within the limits of normality.

Certain findings can increase the likelihood that an otoneurological symptom is related to neurovascular compression. Such findings include deviation of the nerve pathway caused by a vascular structure, vascular compression at the emergence of the nerve root, and a perpendicular intersection between a blood vessel and a nerve^(25)^.

## CONCLUSIONS

Our results show independence between the MRI findings and the clinical profile, suggesting that there is no direct, exclusive relationship between the diagnosis of vascular loop in the AICA identified on an MRI scan and the corresponding otoneurological profile. In view of the current knowledge, we believe that the diagnosis of vascular compression syndrome should not be based only on the findings of the examination (MRI in our case), especially in order to avoid unnecessary or futile interventions.
